# Thymol alleviates AGEs-induced podocyte injury by a pleiotropic effect via NF-κB-mediated by RhoA/ROCK signalling pathway

**DOI:** 10.1080/19336918.2020.1721172

**Published:** 2020-02-07

**Authors:** Qinglian Wang, Zhenwei Shen, Guanghui Qi, Yanfang Zhao, Hongge Zhang, Rong Wang

**Affiliations:** a Department of Nephrology, Shandong Provincial Hospital Affiliated to Shandong University, Jinan, China; b Department of Biostatistics, WuXi Clinical Development Service (Shanghai) Co., Ltd, Shanghai, China; c Department of Urological Surgery, The First Hospital of Zibo, Shandong, China; d Department of Urological Surgery, Tengzhou Hospital of Traditional Chinese Medicine, Zaozhuang, China

**Keywords:** Thymol, advanced glycation end products (AGE), human podocytes, RhoA/ROCK pathway, nuclear factor-kappa B (NF-κB)

## Abstract

Advanced glycation end products (AGE) are those of the most powerful pathogenic factors that related to diabetic complications. In our study, we investigated the beneficial effects of thymol on AGE induced cell injury and apoptosis in human podocytes (HPCs) and attempted to clarify its mechanisms. Our results revealed that stimulation with AGE could significantly activate RhoA/NF-κB pathway. Results showed thymol could markedly suppress inflammatory responses, cell apoptosis and disordered cytoskeleton. Also thymol restored the expression of podocin, restrained migration capacity. Western blot analysis indicated that it could restore the expression of RhoA, ROCK and vimentin, nephrin, podocin and p65 and IκBα phosphorylation. Moreover, si-RhoA also suppressed the expression of pro-inflammatory cytokines, ROCK, and vimentin and the phosphorylation of p65 and IκBα. In conclusion, thymol inhibits AGE-induced cell injury in HPCs by suppressing the RhoA-NF-κB pathway and may be apromising therapeutic agent.

## Introduction

The incidence of diabetes is increasing yearly, and diabetic nephropathy (DN), the most common cause of end-stage renal disease (ESRD) worldwide [1,2], accounts for the high disability and mortality rate in diabetic patients. Approximately 40% of patients with type 1 or 2 diabetes will develop DN in 20–25 years with glomerular sclerosis, which requires much government spending [3]. Although numerous studies have been devoted to prevent, treat and restore the pathogenesis of DN, effective and applicable therapeutic options are far from satisfactory. While intensive blood glucose and blood pressure therapies can be beneficial, it is often difficult to maintain these treatments, and they may increase the risk of hypoglycaemia or hypotension in diabetic patients [4]. Furthermore, some patients will ultimately develop DN even with strict blood glucose and blood pressure control. Therefore, the development of new insights and novel therapeutic strategies that specifically target DN is urgently required.

Advanced glycation end products (AGE) are formed by a nonenzymatic glycation process in which cellular proteins, nucleic acids, and lipids are modified. Several studies have reported that AGE are involved in many different diseases, including DN [5–7]. In the serum of DN patients, the AGE level is much higher due to prolonged hyperglycaemia [7–9]. The pathogenesis of diabetes complications is complex, and a number of factors are involved. Apart from AGE, a microinflammation environment, oxidative stress, the renin-angiotensin system (RAS) and protein kinase C (PKC) all jointly participate in the occurrence and development of DN [10]. According to the results of Busch M. et al., high levels of AGE contribute to a series of pathological changes, including podocytes hypertrophy with cell cycle arrest and apoptosis, increased migration ability and EMT, and the generation of pro-inflammatory cytokines [11]. AGE are thought to play a vital role in the pathogenesis of chronic diabetes complications [12,13]. Vlassara H. reported that treatment of nondiabetic rats with AGE can induce DN and presents as the proteinuria and histological changes similar to DN rats. Furthermore, by preventing the formation of AGE, the occurrence of diabetic complications in animal models is postponed [14].

**Figure 1.**
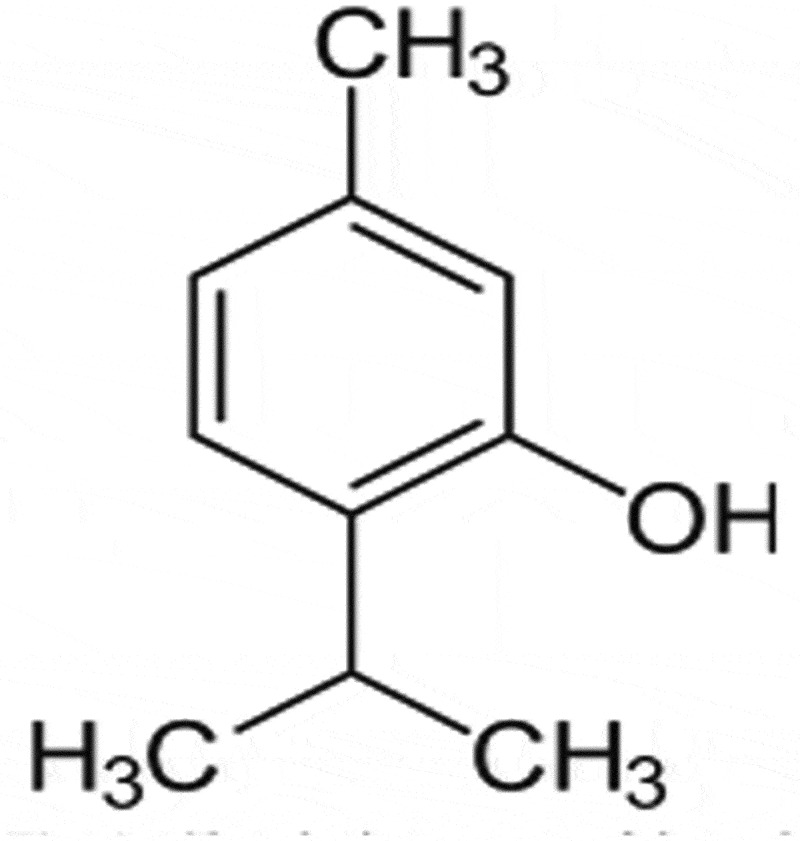
Chemical structure of thymol

Thymol (2-isopropyl-5-methylphenol; Figure 1) has been used in traditional medicine for many centuries and is one of the most important dietary constituents in the thyme species. It is widely used because it possesses pharmacologically relevant antioxidant, anti–inflammatory, antibacterial, antifungal, and antiseptic properties [15]. Relevant studies have revealed that thymol can exert an anti–inflammatory action by suppressing pro-inflammatory signalling pathways and transcription factors such as p38 mitogen-activated protein kinases (MAPK), signal transducer and activator of transcription 3 (STAT-3), NF-κB and activator protein-1 (AP-1) in LPS-induced inflammation [16–20]. In our previous research, we found for the first time that thymol inhibits the inflammatory response induced by LPS in HMrSV5 cells through the TLR4-mediated RhoA-dependent NF-κB signalling pathway [21]. However, the DN-protective effect of thymol in podocytes and the mechanisms underlying these protective effects are not fully understood.

Podocytes are highly differentiated cells in the visceral epithelial cell layer of filtration membranes and have a limited capacity to proliferate and regenerate after injury. Because the glomerular capillary wall functions as an efficient and selective barrier, podocytes are critically involved in maintaining the glomerular filtration barrier. Therefore, podocytes play a critical role in maintaining the filtration membrane integrity. Once these cells are injured, proteinuria associated with DN and the progression of DN occurs [1,22]. Damage to podocytes can lead to proteinuria and initiate glomerulosclerosis, which can result in the progressive loss of renal function [23]. Thus, it is urgently needed to elucidate the mechanisms related to injury in DN and provide therapeutic options to restore injured podocytes.

In the present study, we aimed to clarify the potential protective effects of thymol in AGE-induced podocyte injury to mimic DN changes to investigate the underlying molecular mechanisms.

## Results

### AGE treatment activates RhoA in human podocytes

**Figure 2.**
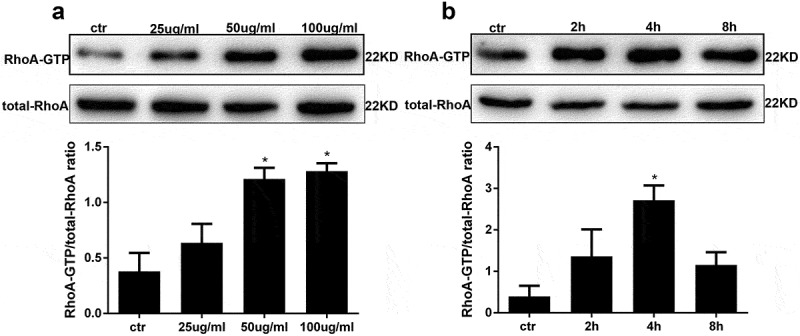
AGE treatment activates RhoA in HPCs. (a) RhoA activation in HPCs treated for 4 h with increasing concentrations of AGE. (b) RhoA pull-down activity assay in HPCs treated with 100 μg/mL AGE over time. (Data are the mean ± S.D.; *P < 0.05 vs control, n = 3)

**Figure 3.**
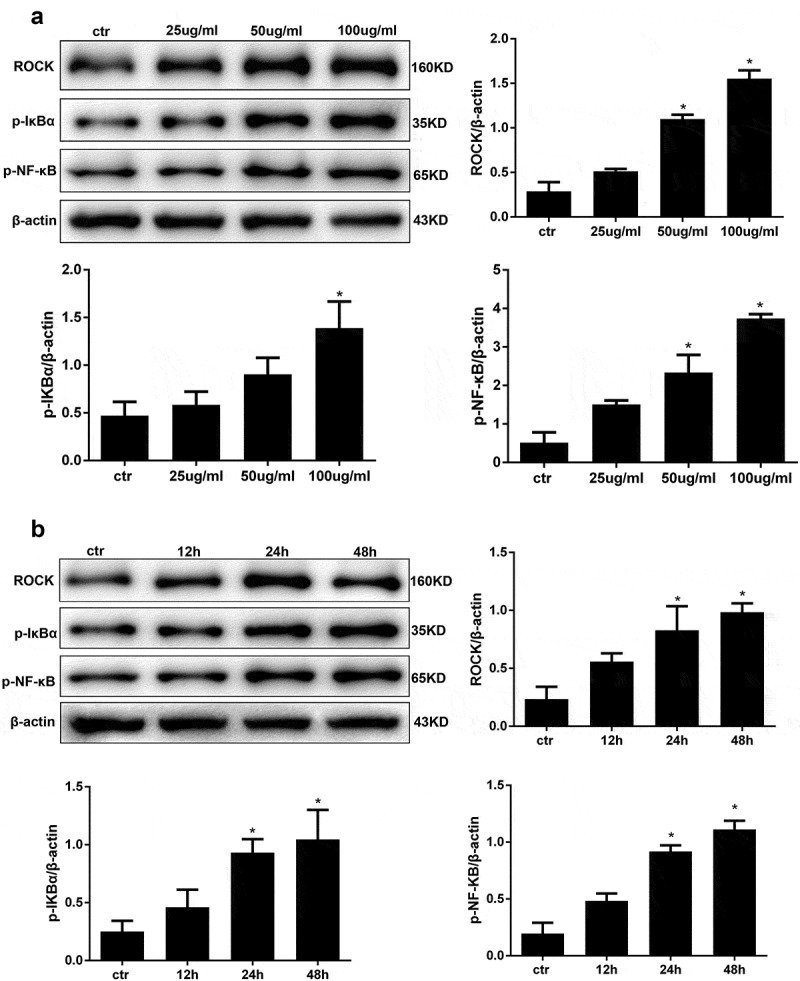
AGE treatment activates ROCK and the NF-κB signalling pathway in HPCs. (a) Detection of ROCK, p-IκBα, p-NF-κB after treatment with increasing concentrations of AGE for 48 h. (b) Detection of ROCK, p-IκBα, and p-NF-κB after exposure to 100 μg/ml AGE over time. (Data are the mean ± S.D.; *P < 0.05 vs control, n = 3)

To assess RhoA activation in AGE-stimulated human podocytes, a RhoA pull-down activation assay was performed. First, we applied an AGE concentration course to human podocytes treated for 4 hours. The results showed that RhoA activity was the highest at 50μg/ml and 100 μg/ml compared with other groups (Figure 2(a)). Combining with the following results analysis (Figure 3(a)), subsequently, podocytes treated with 100 μg/ml AGE for various times showed maximal RhoA activity after 4 hours of treatment (Figure 2(b)).

### AGE treatment activates ROCK and NF-κB in human podocytes

Transcription factor NF-κB is a critical regulator of gene expression during the inflammatory response and in other stress reactions. Hence, we performed western blot analysis to analyse the expression levels of the RhoA downstream molecules ROCK and NF-κB in AGE-stimulated human podocytes. First, we carried out a concentration course and applied an AGE treatment for 48 hours. As expected, the expression level of ROCK was significantly upregulated in a concentration-dependent manner when the cells were treated with AGE compared with the control group (Figure 3(a)). The maximum ROCK activation was found with 100 μg/ml AGE treatment. In addition, the expression levels of p-IkBa and p-NF-κB/p65 were also significantly increased in a concentration-dependent manner after treatment with AGE for 48 h (Figure 3(a)). This indicated that the activity of NF-κB/p65 was also upregulated in human podocytes when stimulated by AGE at 100 μg/ml. Next, time course experiments using this stimulation concentration showed that treatment for 48 hours significantly activated ROCK and NF-κB compared with the control group (Figure 3(b)). Therefore, 48 hours was used as the stimulation time period in subsequent studies.

### Effect of thymol and si-RhoA on human podocyte viability

**Figure 4.**
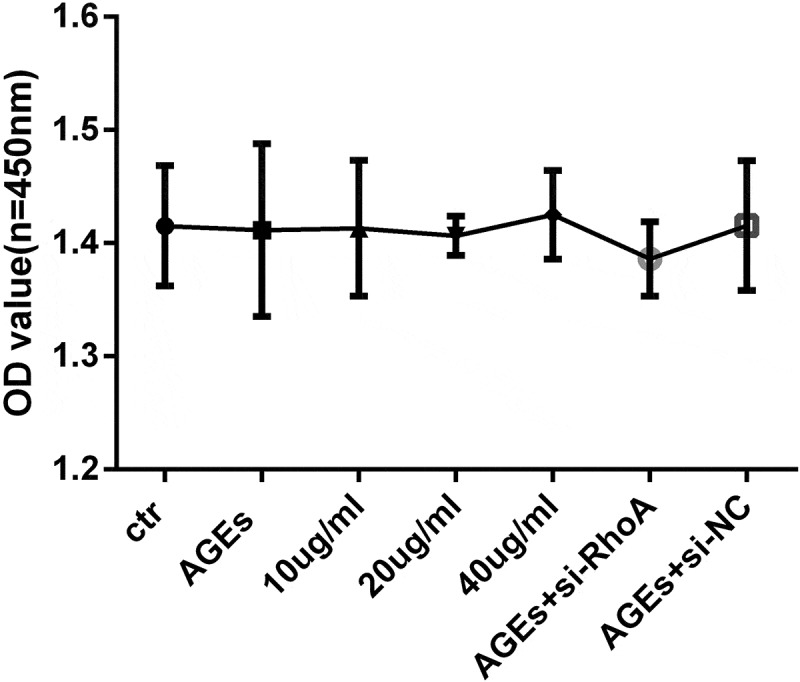
Cell viability assays. HPCs were cultured with different concentrations of thymol, si-RhoA or si-NC in the presence of 100 μg/mL AGE for 48 h. The WST-8 assay showed that there was no significant difference in cell viability among all of the experimental groups. The values are presented as the mean ± S.D. (n = 4)

To assess the potential cytotoxicity of thymol and siRNA on human podocytes, a WST-8 assay was used to detect the viability. The results showed that cell viability was not affected by thymol or si-RhoA administration when combined with AGE (Figure 4).

### Pleiotropic protective effect of thymol on AGE-treated human podocytes

It is well known that there is an inflammation microenvironment in DN patients. Therefore, we investigated the AGE-induced cytokine response to observe the role of thymol. In our experiment, the expression of cytokines TNF-α and IL-6 was detected by ELISA in AGE-treated human podocytes. The results indicated that AGE treatment significantly increased the expression of IL-6 and TNF-α. Moreover, pretreatment with thymol (10, 20, and 40 μg/mL) for 1 h before AGE stimulation decreased the cytokine levels in a dose-dependent manner (Figure 5(a)). Prolonged hyperglycaemia will induce podocyte detachment and apoptosis. In the early stage of diabetes mellitus, podocyte apoptosis and foot process effacement are major pathophysiological features. We also detected the level of apoptosis in different groups by flow cytometry. The results revealed that thymol can efficiently downregulate the apoptosis ratio, which was upregulated in AGE-stimulated cells. These findings demonstrate that thymol inhibits the induction of pro-inflammatory cytokines and apoptosis by AGE (Figure 5(b)).

**Figure 5.**
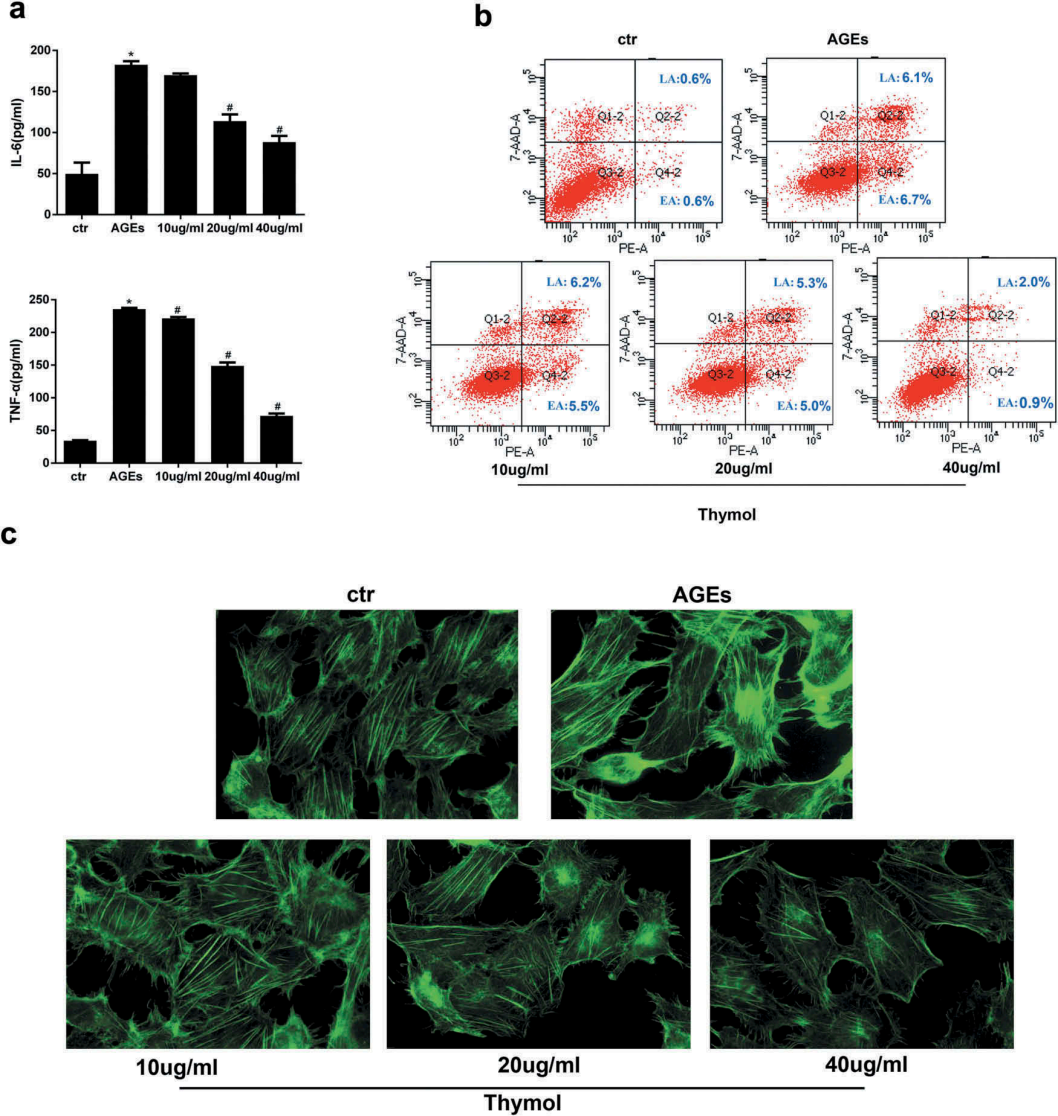
Thymol exhibited pleiotropic protective effects, which were induced by AGE. (a) ELISA indicated that the high expression levels of IL-6 and TNF-α in AGE-stimulated HPCs were restored by thymol in a dose-dependent manner. (Data are the mean ± S.D.; *P < 0.05 vs control, n = 3). (b) The high apoptosis rate that was induced by AGE was restored by thymol in a dose-dependent manner, according to flow cytometry results. EA represents early apoptosis and LA represents late apoptosis. (c) The cytoskeleton of HPCs stained by FITC-phalloidin showed that the rearrangement induced by AGE can be efficiently restored by thymol. (d) The low expression level of podocin in HPCs treated with AGE was significantly rescued by thymol, as detected by IF. (e/f) The cell migration capacity was detected by a wound healing assay (e) and a Transwell assay (f). The results showed that AGE induced a strengthened migration capacity, but this could be alleviated by thymol. (g) Semiquantitative analysis of the wound healing assay (e) and transwell assay (f). (Data are the mean ± S.D.; *P < 0.05 vs control, n = 3)

**Figure 5.**
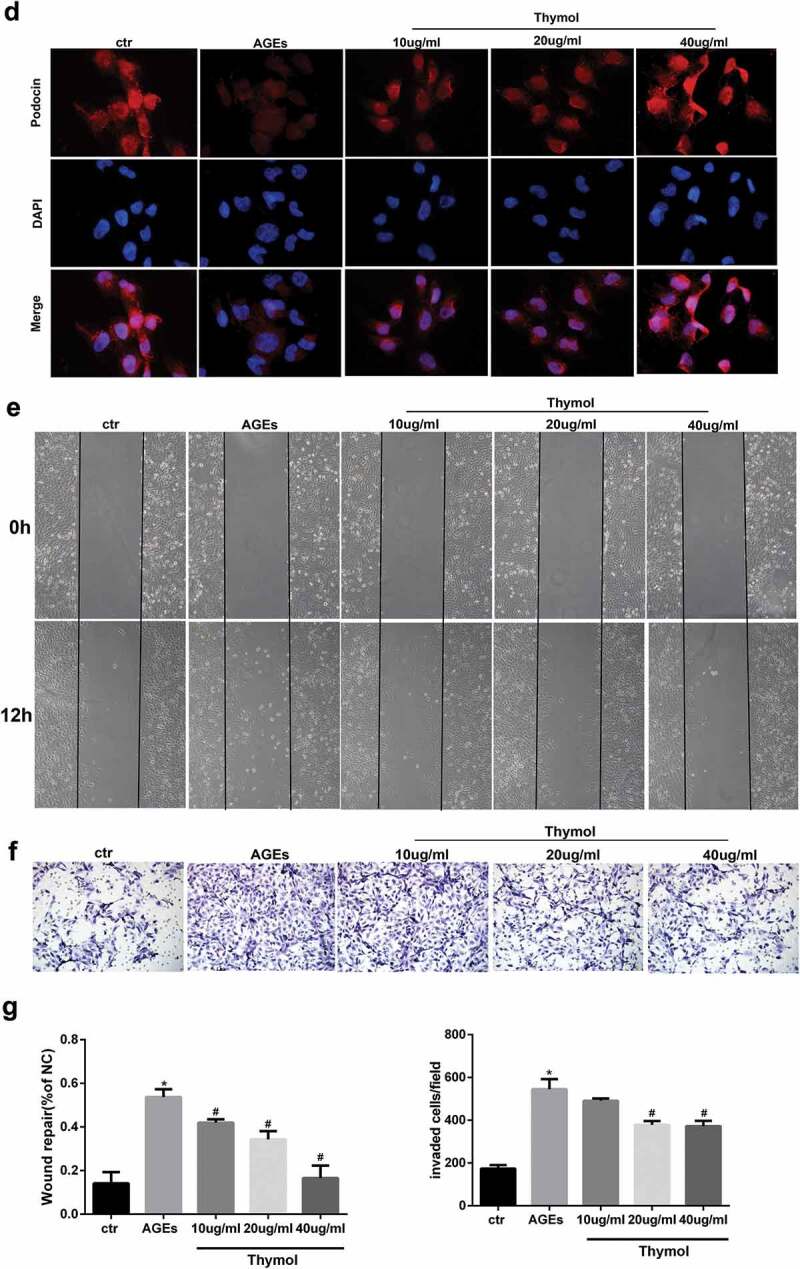
Continued

As an important part of the glomerular filtration barrier, podocytes can prevent the production of proteinuria via the complex regulation of F-actin and the cytoskeleton in the foot process. Fluorescence images of F-actin staining in podocytes showed it to be predominantly distributed in cortical structures around the cell periphery, with only a few thin stress fibres located within the cell body. However, after treatment with AGE for 48 hours, a clear reorganization of F-actin in the cytoskeleton was observed at the cell periphery and in the stress fibre network that spanned the cell body. Furthermore, the stress fibres became dramatically thick and robust, and cells pretreated with thymol could significantly reinstate F-actin assembly, as demonstrated by the decreased staining of F-actin bundles in the submembrane region compared with podocytes subjected to AGE treatment without thymol (Figure 5(c)).

Podocin and nephrin are well-known podocyte-specific protein markers, and vimentin is a characteristic of mature podocytes. Thus, these molecules were used to evaluate podocyte damage. Immunofluorescent staining demonstrated that podocytes cultured in the control group had high expression level of podocin, and its fluorescent intensity was decreased when treated with AGE. However, when the cells were pretreated with thymol, the expression level of podocin was eventually restored (Figure 5(d)).

### Effects of thymol on migration ability in AGE-induced human podocytes

The RhoA/ROCK pathway is reportedly related to the adhesion induced by AGE in glomerular endothelial cells [24]. In the early stage of DN, podocytes acquire motility through the occurrence of EMT, which then slowly disappeared. Therefore, wound healing assays and transwell migration assays were conducted to evaluate AGE-induced motility changes in human podocytes to observe the role of thymol. Compared with the control group, AGE exposure markedly increased cell migration, and this effect was dramatically reversed in the thymol intervention group in a concentration-dependent manner, which can be seen in wound healing assay (Figure 5(e)) and transwell assay results (Figure 5(f)). Taken together, these findings demonstrate that thymol can ameliorate the migration ability in AGE-induced human podocytes.

### Effect of thymol on RhoA and NF-κB signal pathway activation

**Figure 6.**
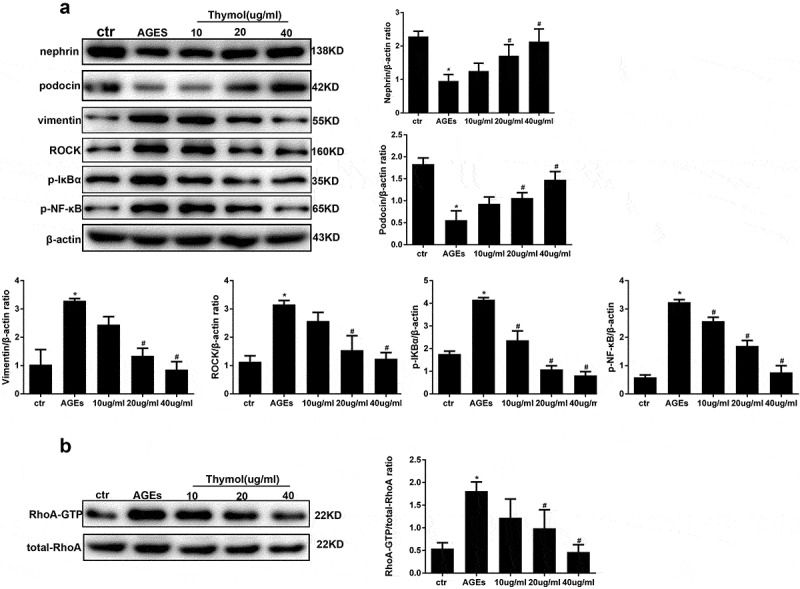
Thymol can reverse the expression of nephrin, podocin and vimentin by inhibiting the RhoA/NF-κB signalling pathway. (a). Nephrin, podocin vimentin and ROCK protein expression and the phosphorylation of NF-κB p65 and IκBα in HPCs were detected by western blot; β-actin served as an internal control. The results showed that thymol could significantly restore the expression of these molecules, which were induced by AGE in a dose-dependent manner. (b) RhoA-GTP was detected by pull-down assay. The results showed that thymol could inhibit it activation. Ctr group indicates the BSA control group; AGE indicates the AGE-stimulated group; 10, 20, and 40 refer to AGE plus 10 μg/ml, 20 μg/ml or 40 μg/ml thymol. (Data are the mean ± S.D.; *P < 0.05 vs control, # *P* < 0.05 vs AGE. n = 3)

To clarify the molecular mechanism of thymol in podocytes in DN, we assessed the effect of thymol on the ROCK and NF-κB signalling pathways, which are activated by AGE. The results indicated that the expression of ROCK, p-IκBα and p-NF-κB/p65 were markedly increased after AGE exposure; however, they were dose-dependently downregulated by thymol administration (Figure 6(a)). Furthermore, we also analysed the activity of RhoA and observed that thymol could significantly inhibit the activation of RhoA (RhoA-GTP decreased) in a concentration-dependent manner, which was previously activated by AGE treatment (RhoA-GTP increased) (Figure 6(b)). The expression of podocin and vimentin were also restored by thymol (Figure 6(a)).

### Effect of si-RhoA on cytokine levels, cytoskeleton rearrangement and migration ability

**Figure 7.**
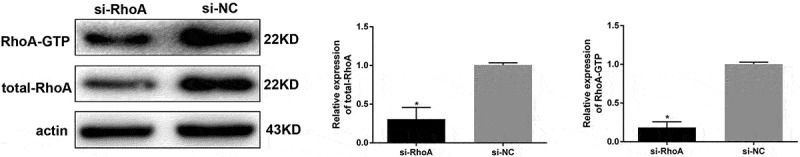
Detection of RhoA silencing effect. The silencing efficiency of si-RhoA was detected by western blot. After RhoA was silenced, the expression of total RhoA and RhoA-GTP were both downregulated. (Data are the mean ± S.D.; *P < 0.05 vs si-NC, n = 3)

**Figure 8.**
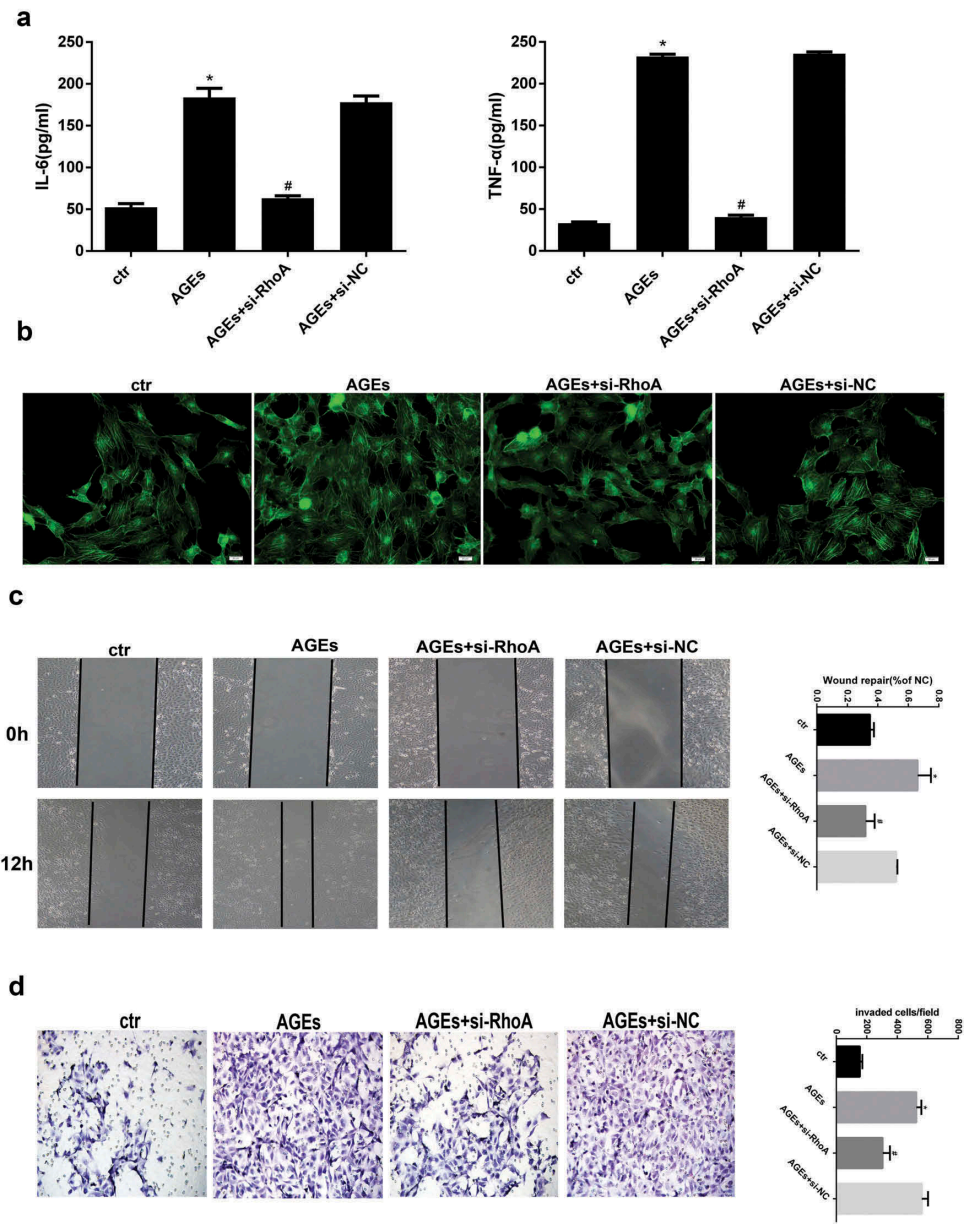
Effects of RhoA silencing on cytokine expression, the cytoskeleton and cell migration capacity. (a) ELISA revealed that the IL-6 and TNF-α levels can be significantly downregulated by si-RhoA. (Data are the mean ± S.D.; *P < 0.05 vs control, #*P* < 0.05 vs AGE. n = 3). (b) Cytoskeleton staining by FITC-phalloidin showed that RhoA silencing can restore disordered cytoskeleton. (c/d) The cell migration capacity was detected by a wound healing assay (c) and Transwell assay (d). The results showed that the strengthened migration capacity could be alleviated by si-RhoA treatment. Ctr indicates the normal control group. AGE indicates the AGE-stimulated group. si-RhoA indicates the AGE plus si-RhoA group, and si-NC indicates the si-NC plus AGE group. (Data are the mean ± S.D.; *P < 0.05 vs control, # *P* < 0.05 vs AGE. n = 3)

To further evaluate whether the protective effect of thymol on the NF-κB signalling pathway was mediated by inhibiting the RhoA pathway, we knocked down RhoA in HPCs using siRNA against RhoA (si-RhoA). We then detected the silencing effect of si-RhoA on the protein level and found that it could significantly decrease the total RhoA and RhoA-GTP expression by more than 70% compared to the si-NC group (Figure 7). After the podocytes were successfully transfected, ELISA revealed that thymol could efficiently ameliorate the expression of inflammatory cytokines IL-6 and TNF-α when the cells were additionally treated with AGE, suggesting that RhoA silencing is central to the anti–inflammatory mechanism of thymol (Figure 8(a)).

Similarly, cytoskeleton staining, wound healing assays and transwell assays were also conducted to detect the key effect of RhoA. The results showed that after RhoA was silenced, F-actin reassembly arrangement was effectively reinstated (Figure 8(b)). Moreover, the cell migration tests also revealed that after si-RhoA transfection, the cell migration ability was clearly decreased. Specifically, the wound healing assay and transwell assay showed that RhoA silencing could decrease the migration ability, as shown in Figure 8(c/d).

### Effect of si-RhoA or ROCK inhibitor on the NF-κB signalling pathway and cell injury

**Figure 9.**
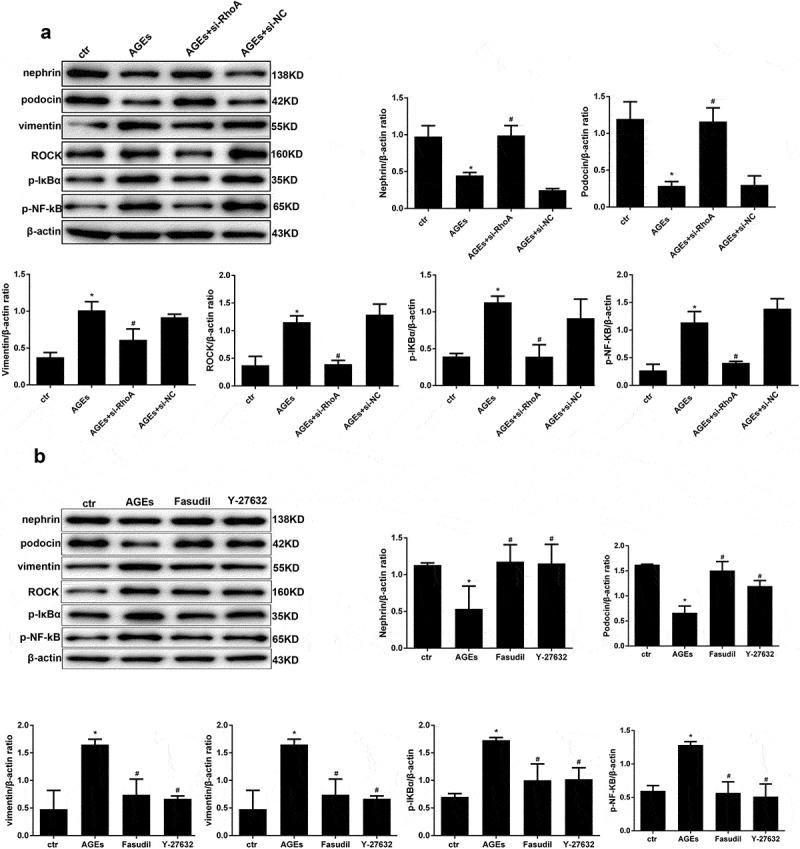
Effect of si-RhoA or ROCK inhibitor on the NF-κB signalling pathway and cell injury. (a) Nephrin, podocin and vimentin protein expression and the phosphorylation of NF-κB/p65, IκBα and ROCK in HPCs were detected by western blot; β-actin served as an internal control. si-RhoA could efficiently restore their expression. (b) The ROCK inhibitor fasudil and Y-27,632 could significantly restore the expression of the above molecules, which were induced by AGE. (Data are the mean ± S.D.; *P < 0.05 vs control, #*P* < 0.05 vs AGE. n = 3)

To further investigate the effect of RhoA in AGE-induced cell injury, WB was conducted. The results revealed that the HPC-specific indexes nephrin and podocin which were downregulated by AGE, could be restored by si-RhoA treatment and the upregulation of vimentin could be restored by si-RhoA treatment also (Figure 9(a)). The effect of RhoA on downstream molecules was also detected by WB. We found that the expression levels of ROCK, p-IκBα and p-NF-κB in the AGE-induced group were significantly upregulated, while they were significantly downregulated in the si-RhoA plus AGE group (Figure 9(a)). To further solidify our conclusions, specific inhibitors of ROCK – fasudil or Y-27632 – were used in our experiment. The results were in line with expectations, as shown in Figure 9(b). These results bolstered our conclusion that the NF-κB signalling pathway is involved in protective effect of thymol and is mediated by RhoA.

## Discussion

DN is a serious complication of diabetes mellitus and is the first pathogenesis of ESRD. Podocytes are critical components involved in maintaining the glomerular filtration barrier; damage to them can lead to proteinuria and eventually progress to glomerulosclerosis, which can result in chronic kidney disease [23]. The concentration of AGE is significantly increased in diabetes mellitus patients, and these compounds function as an accelerator of diabetes and the associated severe complications [25]. Worse still, with renal function impairment, the AGE concentration is further increased and promotes kidney injury, forming a vicious cycle. Thymol is a colourless crystalline monoterpene phenol that has been used in traditional medicine and possesses anti–inflammatory [15] and anti-oxidative stress properties [26]. However, as yet, there are limited data available regarding AGE-induced podocyte injury. In this study, we aimed to explore the protective effect of thymol on AGE-treated podocytes to further establish its related mechanisms.

Exceeding AGE is a strong stimulus for the whole system which can promote our oxidative stress reaction and cell injury. In terms of the kidney, AGE can induce multiple pathophysiological effects for podocytes, including cell hypertrophy, arrest and apoptosis, and reducing cell adhesion and promoting cell migration, along with the generation of inflammatory cytokines. In our study, it was clear that podocytes showed a significantly increased expression level of IL-6 and TNF-α and an increased apoptosis rate upon exposure to AGE. In addition, AGE also promoted cytoskeletal rearrangement and the cellular migration ability, as detected by the wound healing and transwell assays. Finally, the specific markers podocin and nephrin were decreased. It has been previously reported that AGEs can reduce podocyte adhesion via upregulation of ILK expression [27] and can also act as potent stimulators of chemokine production [28]. In diabetes patients, the level of AGE showed a significant increase compared with healthy controls. The tissue levels of AGE in diabetic renal disease have been shown to be twice those of patients with only diabetes and no renal disease [29]. According to recent research, the reduction of the podocytes is an initial change of DN and precedes the development of proteinuria and renal function decline [30,31]. Therefore, it is very important to restore cell function and reduce apoptosis.

The inflammation seen in DN does not merely activate macrophages and neutrophil infiltration; it also affects the podocytes themselves, which are the main source of inflammatory cytokines in response to stimuli [32]. We found that AGE can promote the expression of IL-6 and TNF-α in podocytes by activating the NF-kB pathway. TNF-α is a type of innate immune response component, while IL-6 is a key player in modulating inflammation and is a major effector of the acute phase reaction; it is controlled by TNF-α in peritoneal mesothelial cells (Bellingan et al., 1996). These pro-inflammatory factors play different functional roles in different phases of the inflammation response [33]. In this study, we found that the expression of TNF-α and IL-6 was markedly increased after AGE stimulation. However, pretreatment with thymol inhibited the production of these cytokines in a dose-dependent manner. These results are in accordance with previous findings in RAW 264.7 [19] and mouse mammary epithelial cells [17].

In our research we found that AGE could activate RhoA in a concentration- dependent manner over a rapid time frame. The NF-κB signalling pathway was also activated in a concentration- and time-dependent manner. According to our preliminary results, we induced podocytes for 48 hours with 100 µg/ml AGE in the subsequent experiments. The Rho family of small GTPase proteins, including RhoA, controls a wide variety of cellular processes. RhoA is one of the best-known members of this family. In our previous research, we found that RhoA can be rapidly activated upon stimulation for as little as 30 min [21]. In the current study, we found that podocytes treated with AGE for 2 hours showed a significantly increase in the RhoA-GTP form, while the total amount of RhoA remained unchanged. RhoA acts as a molecular switch that cycles between a GDP-bound form and a GTP-bound form via its intrinsic hydrolytic activity. The Rho kinases are the first and the best-characterized RhoA effectors. Among the critical cellular processes regulated by RhoA/Rho-kinase signalling are inflammation, motility, proliferation, differentiation, and apoptosis [34]. We also found that with increased AGE concentrations and extended incubation times, the expression levels of ROCK, p-IκBα and p-NF-κB-p65 eventually increased. The RhoA/NF-κB axis has been reported in several studies to be responsible for inflammation and apoptosis [35,36]. In our previous research, we also found that LPS activated the NF-κB signalling pathway via RhoA to induce a series of inflammatory reactions.

In the current study, to further investigate the role of RhoA in AGE-induced podocyte damage, siRNA-RhoA was transfected into the podocytes. We found that after RhoA was downregulated, the apoptosis ratio of podocytes was significantly decreased, as were the levels of inflammatory factors TNF-α and IL-6. Previous studies have shown that T2DM patients have a 3-4-fold greater serum level of TNF-α compared with nondiabetic controls and that urinary TNF-α excretion correlates well with the stage and progression of this disease [37]. Previous experimental research also indicates that the level of IL-6 in diabetic kidneys correlates with proteinuria and kidney hypertrophy [38,39]. Nephrin and podocin are two podocyte-specific proteins that are pivotal in constructing the slit membrane and in maintaining an intact filtration barrier [40]. In the present study, the nephrin and podocin expression levels were markedly increased following the transfection of AGE-treated podocytes with siRNA-RhoA. This finding indicates that RhoA mediates podocyte injury and that RhoA downregulation can be a protective factor. In addition, after RhoA was downregulated, the high expression of the NF-κB signalling pathway-related molecules were all restored, at least partly. This clearly shows that RhoA is located upstream of the NF-κB signalling pathway and that the protective effect of thymol on NF-κB signalling is mediated by RhoA.

In conclusion, our study demonstrated that podocytes treated with AGE can activate RhoA and the NF-κB signalling pathway to induce inflammatory reactions, cell apoptosis, and cytoskeletal rearrangement, culminating in cell injury. Thymol exhibited a strong protective effect on podocytes that were treated with AGE. Furthermore, we found that the RhoA-dependent NF-κB signalling pathway is relevant in the protective effect exerted by thymol. Therefore, our findings suggest that thymol could be a potential therapeutic agent for use in patients with diabetic nephropathy.

## Materials and methods

### Chemicals and reagents

A stock solution of 20 mg/ml thymol (endotoxin-free, purity >99.9%; Shanghai Yuanye Bio-Technology Co., Ltd., China) in ethanol was made and stored at −20°C. The working thymol dilutions were used as previously detailed [21]. AGE (Beijing Biosynthesis Biotechnology, China), Y-27632 (Sigma, USA), and fasudil/HA-1077 (Sigma, USA) solutions were prepared in DMSO to produce either 10 mM (AGE) or 100 mM stocks and were stored at −20°C. Working solutions were also prepared as previously detailed [41]. RPMI 1640 medium and foetal bovine serum (FBS) were purchased from Gibco, Invitrogen (Carlsbad, CA, USA), and trypsin/EDTA was purchased from HyClone (Logan, UT, USA). The WST-8 cell proliferation and cytotoxicity assay kit was purchased from Dojindo (Kumamoto, Japan). The RhoA Pull-down Activation Assay Biochem Kit (BK036) was purchased from Cytoskeleton, Inc. (Denver, USA). ELISA kits to assay human cell supernatant TNF-α and IL-6 were purchased from R&D systems (CA, USA). Lipofectamine 2000 reagent was purchased from Invitrogen (Carlsbad, CA, USA). Primary antibodies against p-IκBα, vimentin and p-NF-κB-P65 were purchased from Cell Signalling Technology (CST, Beverly, USA). Antibodies against Rho-kinase, nephrin and podocin were purchased from Abcam (Abcam, UK). Other antibodies were obtained from Proteintech Biotechnology (Wuhan, China). β-actin and horseradish peroxidase (HRP)-conjugated goat anti-rabbit and goat anti-mouse antibodies were provided by Beyotime Biotechnology (Shanghai, China). The Annexin V-PE kit was purchased from BD Biosciences (Franklin Lakes, NJ, USA). Fluorescent phallotoxin (P5282) and Delight 594 goat anti-rabbit were purchased from Sigma-Aldrich (St. Louis, MO, USA). All other chemicals were of reagent grade and endotoxin free.

### Cell culture

Human podocytes were kindly obtained from professor Fanyi (Department of Pharmacology, Shandong University School of Medicine) as a gift, and we express our sincere appreciation. Briefly, human podocytes were maintained in RPIM 1640 medium supplemented with 10% FBS, 1% penicillin, 1% streptomycin and 10 U/ml interferon-γ in a humidified incubator with 5% CO2 at 33°C to induce proliferation. Before stimulation, human podocytes were transferred to a 37°C incubator and maintained for at least two weeks to induce differentiation. At this time interferon-γ should be moved away. Confluent cells were rendered quiescent by incubation for 24 hour in serum-free medium before treatment with AGE (50 μg/ml) or BSA (50 μg/ml; osmotic control) for various times, as indicated. The Rho-kinase inhibitors Y-27632 (10 μmol/l) and fasudil/HA-1077 (25 μmol/l) were added 30 min before the addition of AGE. The inhibitory activity and specificity of both inhibitors towards Rho-kinase have been previously demonstrated [42].

### RNA interference

Small interference RNA (siRNA) targeting RhoA (si-RhoA: 5ʹ-UCAAGCAUUUCUGUCCCAA-3ʹ) and the negative control RNA probe (NC: 5ʹ-AGUUCAACGACCAGUAGUC-3ʹ) were designed and purchased from Takara (Dalian, China). Human podocytes were seeded onto 6-well plates. When reached approximately 60–70% confluency, they were transfected with Opti-MEM medium containing siRhoA/NC and Lipofectamine™ 2000 for 6 h. The medium was changed and the cells were grown further with fresh RPMI medium for another 24 h. The transfected podocytes were then treated with AGE (100 μg/ml) for 48 hour. The non-transfected cells grown in normal culture medium served as controls. The knockdown efficiency of si-RhoA was then detected.

### WST-8 assays

The cell viability of all the control and experimental groups was evaluated (control, AGE, AGE plus thymol (10, 20, 40 μg/ml), si-RhoA plus AGE, and NC plus AGE) using the WST-8 cell proliferation and cytotoxicity assay kit. Briefly, siRhoA-, NC- and nontransfected podocytes were seeded into a 96-well plate and treated with AGE and thymol (as described previously) in 90 μl of complete medium for 24 hour. Subsequently, 10 μL of WST-8 solution was added to each well and incubated for an additional 2 hour at 37 °C. The absorbance was measured at 450 nm using a microplate reader. The cell viability of the experimental groups was normalized against that of the untreated cells.

### RhoA pull-down assay

The RhoA pull-down assay was performed using a kit according to the manufacturer’s instructions. Briefly, cell lysates were rapidly harvested on ice and centrifuged at 15,000 rpm for 5 min at 4°C. A total of 20 μl of the lysate was saved for protein quantitation, and equivalent amounts of lysate protein (500 μg total protein) were incubated with a predetermined amount of rhotekin-RBD beads at 4°C for 60 min with gentle rocking, followed by centrifugation at 15,000 rpm for 60 s at 4°C. The beads were washed and boiled for 2 min in 20 μl of 2× Laemmli sample buffer. The supernatant was resolved with 12% SDS-PAGE. Membranes were probed with anti-RhoA antibody (1 μg/ml; provided in the kit) and an HRP-conjugated secondary antibody. Protein bands were imaged using the ECL system.

### Western blot analysis

Total protein extracted by RIPA buffer was used to detect the expression levels of all molecules. General use protease and phosphatase inhibitor cocktails (50×, Beyotime Biotechnology, Shanghai, China) were added to the lysis buffer for protein extraction. The proteins (10 μg) were separated with 10–12% SDS-PAGE, and the separated proteins were transferred to polyvinylidene fluoride (PVDF) membranes. The membranes were then blocked with 5% nonfatty milk for 1 hour at room temperature and then incubated at 4°C overnight with the following primary antibodies: rabbit anti-Rho-kinase (1:1000; Abcam, UK), rabbit anti–vimentin (1:1000; CST), rabbit anti-p-NF-κB-P65 (1:1000; CST), rabbit anti-p-IκBα (1:1000; CST), rabbit anti-nephrin (1:1000; Abcam, UK), rabbit anti-podocin (1:1000; Abcam, UK), and rabbit anti-β-actin (1:1000; BOSTER, China). Next, the membranes were washed with TBST buffer and incubated with HRP-conjugated anti-rabbit IgG secondary antibodies (1:5,000; Santa Cruz Biotechnology, Inc. Dallas, TX, USA) for 1 hour at room temperature. Protein bands were detected using an ECL system and a Bio-Rad electrophoresis image analyser (Bio-Rad, Hercules, CA, USA). β-actin was used as internal total protein control.

### Immunofluorescence

Podocytes grown on glass coverslips were pretreated according to the experimental conditions. They were then fixed with 4% paraformaldehyde for 20 mins. After incubation with goat serum working solution to block non-specific binding for 2 hour in 37°C, the cells were incubated with podocin primary antibody at 4°C overnight. The cells were then stained with DyLight 594-conjugated secondary antibodies for 1 hour at room temperature. The cytoskeleton was stained with FITC-conjugated phallotoxin. The cells were incubated with 4,6-diamidino-2-phenylindole (DAPI) for 5 min to stain the nuclei. Randomly selected fields were imaged using a fluorescence microscope.

### Enzyme-linked immunosorbent assay (ELISA)

Podocytes were seeded in 6-well plates (4 × 10^5^ cells/well) and pretreated with or without various concentrations of thymol (10, 20, 40 μg/mL) for 1 h. The cells were then stimulated with AGE (100 μg/mL) for an additional 24 h. The supernatant was collected and centrifuged at 1500 rpm for 3 min to measure the levels of TNF-α and IL-6 using ELISA kits according to the manufacturer’s instructions. The absorbance was measured at 450 nm using a microplate reader. The TNF-α and IL-6 concentrations were calculated according to an absorbance standard curve.

### Wound healing assays

Cells were seeded into 6-well plates and allowed to grow to 90% confluence. A sterile 20 μl micropipette tip was used to scratch the cell monolayers. The cells were then washed with 1 ml of PBS and supplemented with 2 ml of serum-free DMEM-F12, and incubated with AGE and Rho inhibitors as described above. Pictures of migrating cells were taken at 0 h and 12 h. The migratory rates were calculated as (A-B)/A*100%, where A and B reflect the width of the wound at 0 h and 12 h, respectively.

### Transwell migration assays

Transwell cell culture inserts (pore size 8 μm; Corning Co-Star Corp., Cambridge, MA, USA) were placed in RPMI 1640 medium supplemented with 15% foetal bovine serum in the lower compartment. Podocytes with different pretreatments were harvested with trypsin and resuspended in serum-free medium. The upper chambers were seeded with 1 × 10^5^ cells/ml, which were allowed to attach at 37°C for 12 h. Non-migratory cells were then removed from the upper surface of the chambers, and migrated cells on the lower membrane surface were fixed with 4% paraformaldehyde and stained with haematoxylin. The number of migrated cells was counted in 3 separate fields per membrane at 200× using phase-contrast microscopy (Leica Microsystems, GmbH). The data are presented as the mean ± S.D.

### Flow cytometry

Human podocytes were seeded into 6-well plates and treated as previously described. The detection of apoptosis was accomplished with a PE-7-AAD Apoptosis Detection Kit (BD Biosciences, Franklin Lakes, NJ, USA). The staining protocol was performed according to the manufacturer’s instructions. Briefly, the cells were collected with non-EDTA trypsin and centrifugation at 1000 rpm for 10 min. After washing three times with cold PBS, the cells were resuspended by 1× binding buffer at a concentration of approximately 1 × 10^5^ cells/ml. Next, 500 μL of solution was transferred to a 5 ml culture tube to which 5 μl of PE and 5 μl of 7-AAD were added. The cells were incubated in the dark at room temperature for 15 min, followed by detection within 1 h using a FACSVantage SETM flow cytometer (BD Biosciences). Staining with PE Annexin V is typically used in conjunction with a vital dye such as 7-Amino-Actinomycin (7-AAD) to allow the investigator to identify early apoptotic cells (7-AAD negative, PE Annexin V positive). Viable cells with intact membranes exclude 7-AAD, whereas the membranes of dead and damaged cells are permeable to 7-AAD. Cells that are considered viable are PE Annexin V and 7-AAD negative; cells that are in early apoptosis are PE Annexin V positive and 7-AAD negative; and cells that are in late apoptosis or already dead are both PE Annexin V and 7-AAD positive. So we can easily concluded Q4-2 quadrant contains early apoptotic cells and Q2-2 quadrant contains late apoptosis or already dead cells.

### Statistical analysis

The data are presented as the mean ± S.D. unless otherwise stated. One-way ANOVA was used to determine statistically significant differences between groups. Dunnett’s test was used to perform multiple comparisons between groups. A two-tailed P < 0.05 was considered to be statistically significant. The statistical analysis was performed using SPSS 20.0 software (SPSS Inc. Chicago, Illinois, USA).
